# Investigating the mechanism of Tongqiao Huoxue decotion in the treatment of allergic rhinitis based on network pharmacology and molecular docking: A review

**DOI:** 10.1097/MD.0000000000033190

**Published:** 2023-03-10

**Authors:** Fang Zhang, Jiani Wu, Qu Shen, Zhiling Chen, Zukang Qiao

**Affiliations:** a The First Clinical Medical College of Zhejiang Chinese Medicine University, Hangzhou, China; b Department of Otorhinolaryngology, Hangzhou TCM Hospital Affiliated to Zhejiang Chinese Medical University, Hangzhou, China; c Department of Tuina, The First Affiliated Hospital of Zhejiang Chinese Medical University (Zhejiang Provincial Hospital of Chinese Medicine), Hangzhou, China.

**Keywords:** allergic rhinitis, molecular docking, network pharmacology, Tongqiao Huoxue decoction

## Abstract

Allergic rhinitis is prone to recurrence, and clinical treatments focus on control symptoms; however there is no radical cure. Our aim was to use network pharmacology and molecular docking to reveal the hub genes, biological functions, and signaling pathways of Tongqiao Huoxue decoction against allergic rhinitis. First, the chemical components and target genes of Tongqiao Huoxue decoction were obtained from the Traditional Chinese Medicine Systems Pharmacology database. Similarly, allergic rhinitis targets were screened using online Mendelian Inheritance In Man and GeneCards database. Then, all potential targets of Tongqiao Huoxue decoction in the treatment of allergic rhinitis were identified, the Venn diagram was portrayed using R software, and protein-protein interaction network was built using String. The hub genes were analyzed using enrichment analyses. Finally, molecular docking was used to verify the reliability of the key gene prediction. The core targets for Tongqiao Huoxue decoction to improve allergic rhinitis were AKT1, TP53, IL6, and so on. The enrichment analysis results showed that Tongqiao Huoxue decoction treatment in allergic rhinitis might be involved in the AGE-RAGE signaling pathway and fluid shear stress and atherosclerosis pathway. The molecular docking verification indicated that its ingredients bound well to the core targets of allergic rhinitis, and stigmasterol’s docking ability with TNF (−12.73 kcal/mol) is particularly prominent. Based on these findings, it may be deduced that stigmasterol treated allergic rhinitis by acting on TNF targets. But, this conclusion needs to be confirmed by further in vitro and in vivo trials.

## 1. Introduction

Allergic rhinitis (AR) is a nasal mucosa atopic disorder mediated by immunoglobulin E (IgE) and is characterized by nasal pruritus, clear rhinorrhea, sneezing, nasal congestion, post-nasal drip, and pale discoloration of the nasal mucosa.^[1]^ The incidence of AR is 19% among adults and 22% among children in China, and boys showing a higher prevalence than girls.^[2,3]^ The global prevalence of AR is between 10% and 40%.^[4]^ AR as a chronic disease what does not cause death, but it imposes a heavy socioeconomic burden on patients and seriously impairs their quality of life.^[5,6]^ Although the etiology of AR is still unclear, it is environmental, genetic, and epigenetic that strongly associated with onset of AR according to some current reports.^[7]^ AR can be triggered while exposed to allergens including pollen, mold, dust, egg, seafood, and soybean, et al.^[8]^ Health education for patients, irritant and allergen avoidance measures, pharmacotherapy, allergen immunotherapy, biologics, nasal irrigation, acupuncture and surgery are present common therapeutic measures for patients of AR. But there are no radical cure for AR, and clinical treatments are mainly used to control symptoms of AR.^[9]^ Moreover, the efficacious of traditional Chinese medicine (TCM) against AR have been proven and TCM treatment without obvious adverse effects.^[10]^ Tongqiao Huoxue decoction (THD), originated from “Corrections on the errors of medical works” written by the Qing Dynasty physician Wang Qingren is a traditional Chinese prescription, and it’s ingredients include 6 grams of red red peony, 6 grams of Chuanxiong, 6 grams of safflower, 9 grams of peach kernels, 9 grams of ginger 0.15 grams of musk, and 7 red dates. The main therapeutic effect of THD is to improve local blood circulation by dispersing blood stasis and dredging collateral. TCM theory believes that nose is connected to the brain, and the level of interleukin-6 in the blood serum closely relating with the increased occurrence of AR^[11]^ can significantly be reduced by THD. Therefore, THD can be used to treat nasal diseases and the addition and subtraction of Chinese medicine drugs was carried out on the basis of THD in this study.^[12]^ Through the summary of clinical treatment experience, it is found that some Chinese medicines such as comfrey, madder and lnk lotus have excellent efficacy in the treatment of AR. Based on this discovery, we added and subtracted the composition of THD to formulate a new THD, which includes astragalus, codonopsis ginseng, poria, Baizhu, Chuanxiong, red peony, madder, comfrey, lnk lotus, licorice, and the effective of ingredients modified THD has been clinically proven. However the drug active mechanisms of ingredients modified THD in the treatment of AR are still unclear and need to be further explored.

In this research, network pharmacology was firstly applied to identified the active ingredients and targets of THD in the treatment of AR. Next, the protein-protein interaction (PPI) network analysis and enrichment analysis were used to predict the critical molecules of THD for AR treatment. Lastly, molecular docking was performed to reveal molecular regulatory mechanisms in a high-throughput manner.^[13]^

## 2. Materials and methods

### 2.1. Screening active components of THD

The active ingredients of THD were collected through the Traditional Chinese Medicine Systems Pharmacology (TCMSP, http://tcsmpw.com/tcsmp.php) Database. Oral bioavailability (OB) ≥ 30% and drug-likeness (DL) ≥ 0.18 were set as the thresholds for screening the active components in AR. Then, the targets of these active ingredients were obtained from this website. The aggregated targets were input into Uniprot (http://www.uniprot.org/) to obtain gene symbols and gene IDs.

### 2.2. Collecting the disease-related targets

“Allergic Rhinitis” was used as the keyword to search all the possible targets against AR in the online Mendelian Inheritance in Man database (http://www.omin.org/), and GeneCards database (relevance score ≥ 2) (http://www.genecards.org/). The AR-related targets from different databases were merged and dereplicated. Finally, these targets were unified as gene names on UniProt.

### 2.3. Venn diagram of targets between drugs and disease

The intersection target genes between both AR and THD were obtained through Venn 2.1 (http://bioinfogp.cnb.csic.es/tools/venny/). The coincident target genes were displayed in the overlapping domain after amalgamation and striking out the duplicates.

### 2.4. Analysis of PPI network

PPI network of AR and THD reclosing target genes was depicted using the STRING database (http://string-db.org/). According to the corresponding calculation method the species was set as “Homo sapiens” and the threshold was set as 0.9 to show the PPI network. PPI network was visualized in Cytoscape 3.9.1 software and algorithms of cytoHubba plug-in: the Degree Value were used to sift the top 10 hub targets form the network.

### 2.5. Construction of drug-compound-target genes network

The screened ingredients and the overlapping target genes were utilized to build the active component-target-AR network using Cytoscape 3.7.3 software. In addition, the top six components in the Degree value of THD were selected to dock with key genes.

### 2.6. Functional enrichment analyses

The Gene ontology (GO) and Kyoto encyclopedia of genes and genomes (KEGG) pathway enrichment analyses were performed using Bioconductor package and clusterProfiler package in R 4.2.1 software under the condition of *P* < .05 and *q* < 0.05. Finally, the results were presented in bar and bubble graphs.

### 2.7. Molecular docking

To explore the relationship and action mechanisms between candidate proteins and active ingredients, molecular docking simulations were conducted to evaluate the strength and mode of interactions between components and hub targets. Crystal structures of critical targets protein receptors were acquired from the Protein Data Bank database (http://www.rcsb.org/) in Protein Data Bank format. The active component structure as ligands was downloaded from the PubChem compound database (http://pubchem.ncbi.nlm.nih.gov/). After removing the water molecules and organic compounds from ligands and proteins and adding non-polar hydrogen bridge to them by PyMol 2.6.0 software, the format of the molecular ligands and proteins was transformed into pdbqt format. Subsequently, the docking of ligands and proteins was performed by AutoDockTools 1.5.7 software. Each group of molecular docking was run 50 times, and the ionization energy was calculated. The minimum energy value was selected as the docking affinity. Finally, the docking results were visualized using PyMol software.

## 3. Results

### 3.1. Active compounds in THD and candidate targets

232 potential active ingredients of THD were retrieved form TCMSP database under the screening conditions of OB ≥ 30% and DL ≥ 0.18. Since THD contains a total of 11 flavors of Chinese medicine, and there many active ingredients in each flavor of Chinese medicine, only the two active ingredients with the highest OB in each flavor of Chinese medicine are listed (Table 1). 153 corresponding underlying target genes with THD were collected form TCMSP and a sum of 814 for AR were identified from the GeneCards and OMIM.

**Table 1:** Top 2 active ingredients with OB value of each herb in THD.

Mol ID	Herbs	Active compounds	OB	DL
MOL000442	astragalus	1,7-Dihydroxy-3,9-dimethoxypterocarpene	109.99	0.48
MOL000439	astragalus	Isomucronulatol-7,2’-di-O-glucosiole	74.69	0.62
MOL008411	codonopsis ginseng	11-Hydroxyrankinidine	65.95	0.66
MOL008407	codonopsis ginseng	(8S,9S,10R,13R,14S,17R)-17-[(E,2R,5S)-5-ethyl-6-methylhept-3-en-2-yl]-10,13-dimethyl-1,2,4,7,8,9,11,12,14,15,16,17-dodecahydrocyclopenta[a]phenanthren-3-one	65.9	0.76
MOL000273	poria	(2R)-2-[(3S,5R,10S,13R,14R,16R,17R)-3,16-dihydroxy-4,4,10,13,14-pentamethyl-2,3,5,6,12,15,16,17-octahydro-1H-cyclopenta[a]phenanthren-17-yl]-6-methylhept-5-enoic acid	44.17	0.81
MOL000275	poria	trametenolic acid	43.51	0.8
MOL000020	Baizhu	12-senecioyl-2E,8E,10E-atractylentriol	63.37	0.22
MOL000021	Baizhu	14-acetyl-12-senecioyl-2E,8E,10E-atractylentriol	62.4	0.31
MOL001494	Chuanxiong	Mandenol	68.96	0.19
MOL002135	Chuanxiong	Myricanone	65.95	0.51
MOL004355	Red peony	Spinasterol	65.33	0.76
MOL000449	Red peony	Stigmasterol	65.08	0.76
MOL007735	comfrey	Des-O-methyllasiodiplodin	75.08	0.2
MOL002883	comfrey	Ethyl oleate (NF)	73.09	0.19
MOL003283	madder	(2R,3R,4S)-4-(4-hydroxy-3-methoxy-phenyl)-7-methoxy-2,3-dimethylol-tetralin-6-ol	102.89	0.39
MOL000358	madder	beta-sitosterol	77,12	0.75
MOL001790	lnk lotus	Linarin	72.13	0.71
MOL001689	lnk lotus	acacetin	69.94	0.24
MOL001484	licorice	Inermine	90.78	0.54
MOL001792	licorice	DFV	83.71	0.18

DL = drug-likeness, OB = oral bioavailability, THD = Tongqiao Huoxue decoction.

### 3.2. Intersective targets between THD and AR

**Figure 1.**
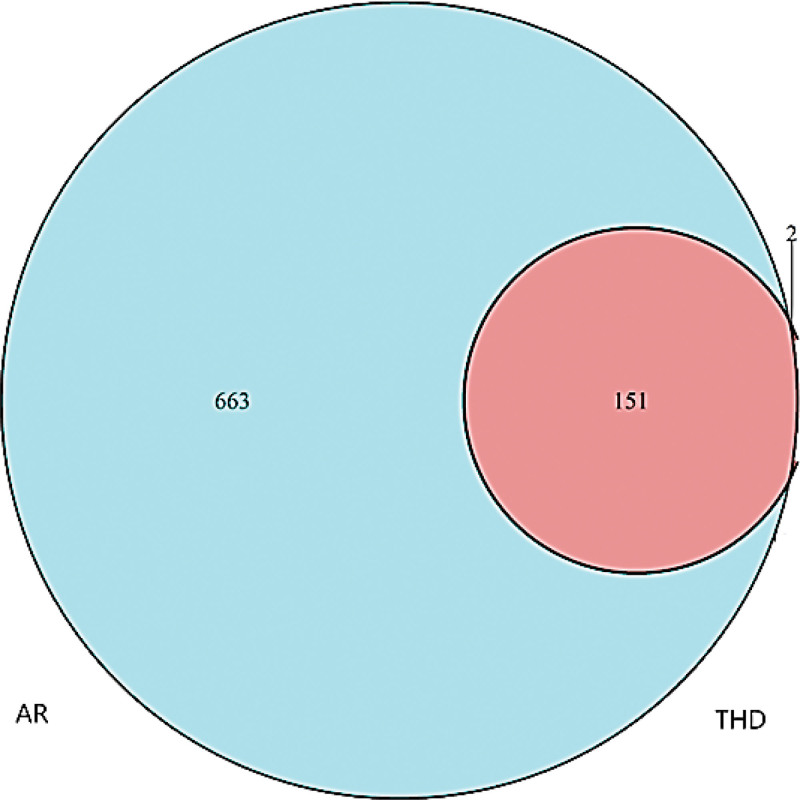
Venn diagram of the interactive targets of THD and AR. The cyan circle represents the targets genes of AR, the red section represents the target genes of THD, and the intersection of the two circles represents the target genes THD for AR. AR = allergic rhinitis, THD = Tongqiao Huoxue decoction.

The 153 ingredients-related target genes and 814 AR-associated targets were imported into the Venn 2.1 software to draw the Venn diagram (Fig. 1). Among them, 151 were common targets of THD and AR.

### 3.3. Drug-compound-target network

**Figure 2.**
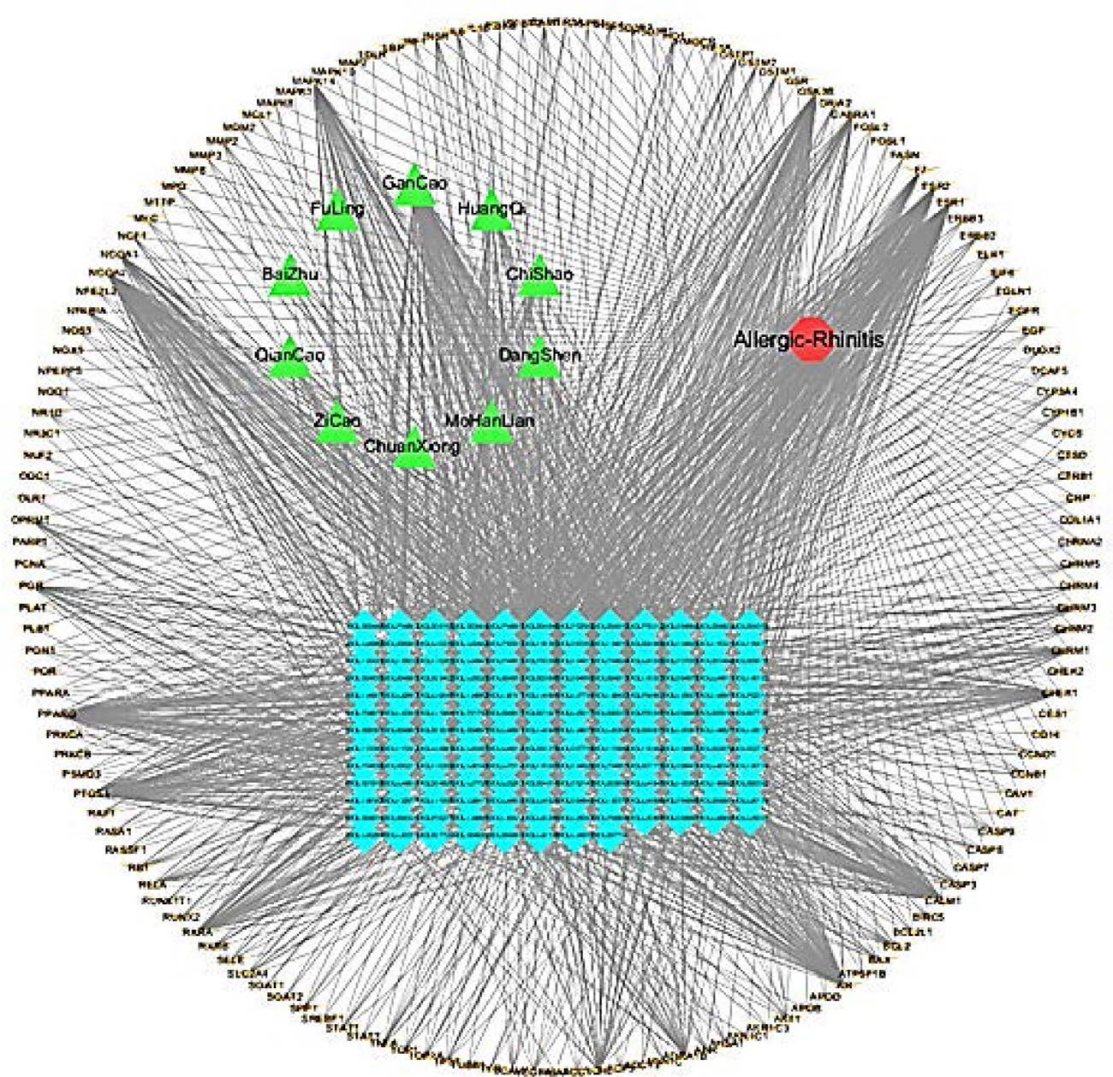
The drug-compound-target network of THD and AR, HQ: astragalus, DS: codonopsis ginseng, FL: poria, BZ: Baizhu, CX: Chuanxiong, CS: red peony, ZC: comfrey, QC: madder, MHL: lnk lotus, GC: licorice. AR = allergic rhinitis, THD = Tongqiao Huoxue decoction.

The candidate targets and corresponding compounds were analyzed by Cytoscape 3.7.3 software to construct a drug-compound-target network to show the relationship between them more intuitively (Fig. 2). The “network analyzer” function in the Cytoscape software was used to perform network topology analysis. according to the degree value, the top five key components of THD treatment AR are ethyl oleate (NF), mandenol, perlolyrine, spinasterol, and stigmasterol.

### 3.4. Analysis of protein-protein interaction network and screening hub targets

**Figure 3.**
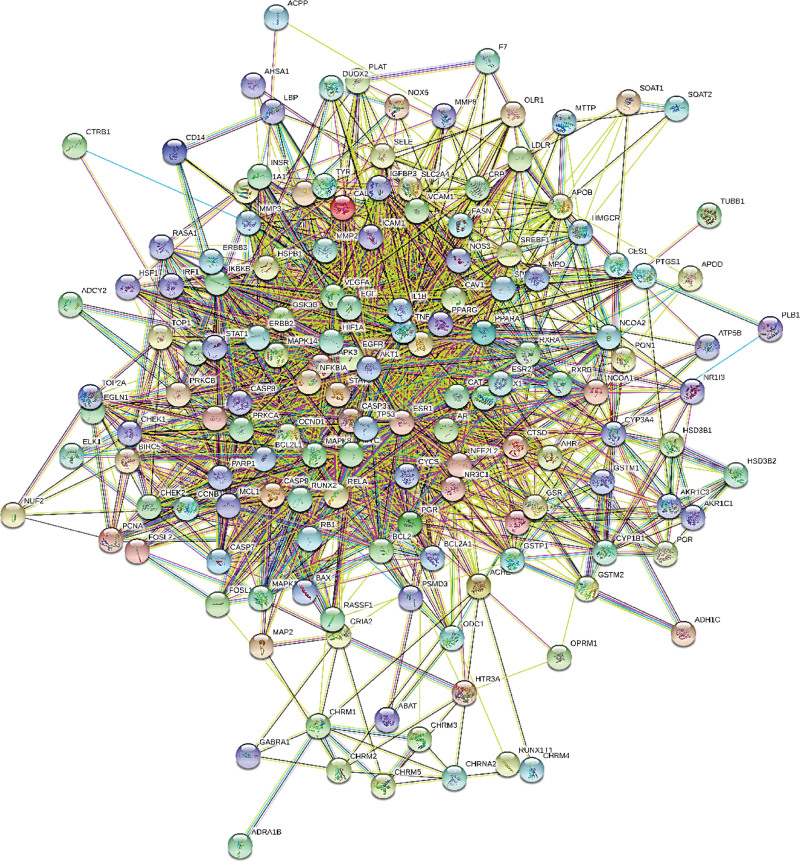
The PPI network. PPI network explored the complex interactions among these 151 intersectant target genes of THD and AR. AR = allergic rhinitis, PPI = protein-protein interaction, THD = Tongqiao Huoxue decoction.

**Figure 4.**
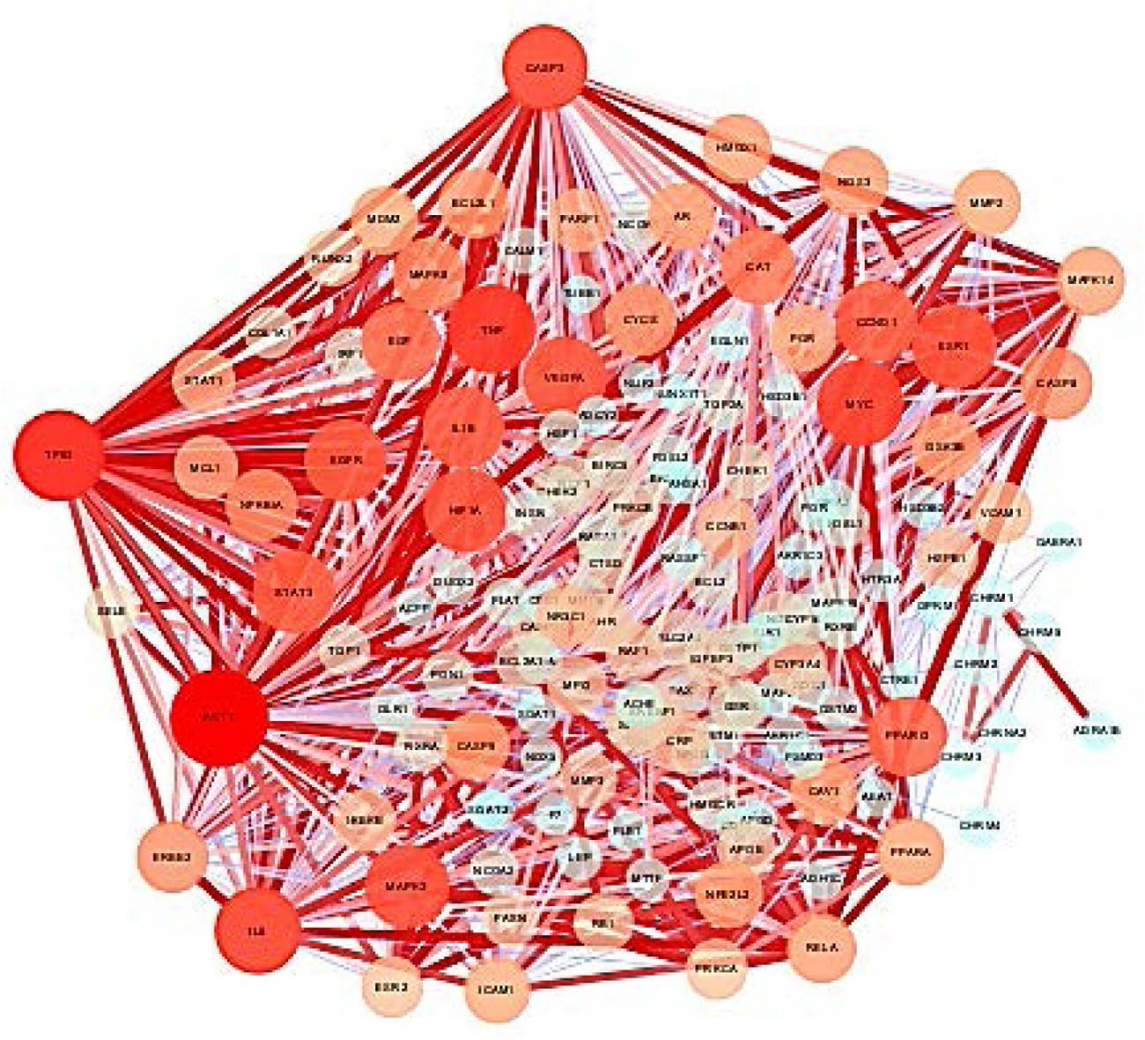
Network diagram of the hub proteins of THD against AR. The darker the color and the larger the size of the node, manifested the higher Degree value. The top 10 genes in terms of degree value are AKT1, TP53, IL6, TNF, CASP3, MAPK3, HIF1A, VEGFA, ESR1, MYC. AR = allergic rhinitis, THD = Tongqiao Huoxue decoction.

The PPI network derived from STRING database was plotted to explore the complex interactions among these 151 intersectant target genes of THD and AR. Subsequently, the PPI network of interactive proteins was inputted into Cytosacpe software for visualization. The core target proteins of THD therapeutic AR were calculated by count screening, as shown in Figures 3 and 4. The interaction of target proteins and these proteins was presented by nodes and edges, respectively. Besides, the darker the color and the larger the size of the node, manifested the higher Degree value (the connectivity between nodes). The top 10 in terms of degree value are AKT1, TP53, IL6, TNF, CASP3, MAPK3, HIF1A, VEGFA, ESR1, MYC. It indicated that these targets mentioned above played an important role in THD in the treatment of AR.

### 3.5. GO and KEGG enrichment analysis

**Figure 5.**
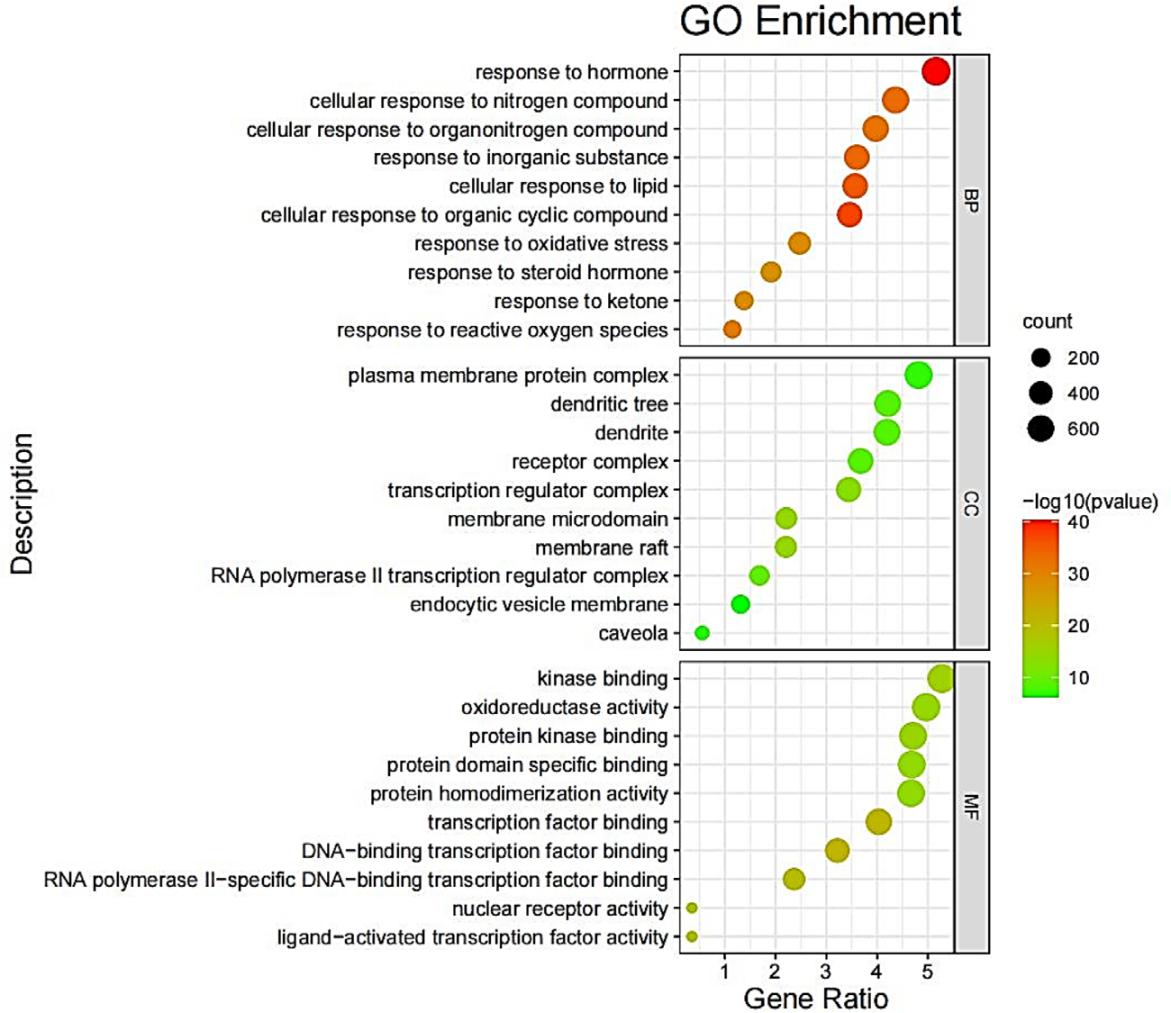
the babble chart of GO enrichment analysis. The most significant 10 results of GO functional enrichment analysis in terms of biological process, cellular component, and molecular function. GO = gene ontology.

**Figure 6.**
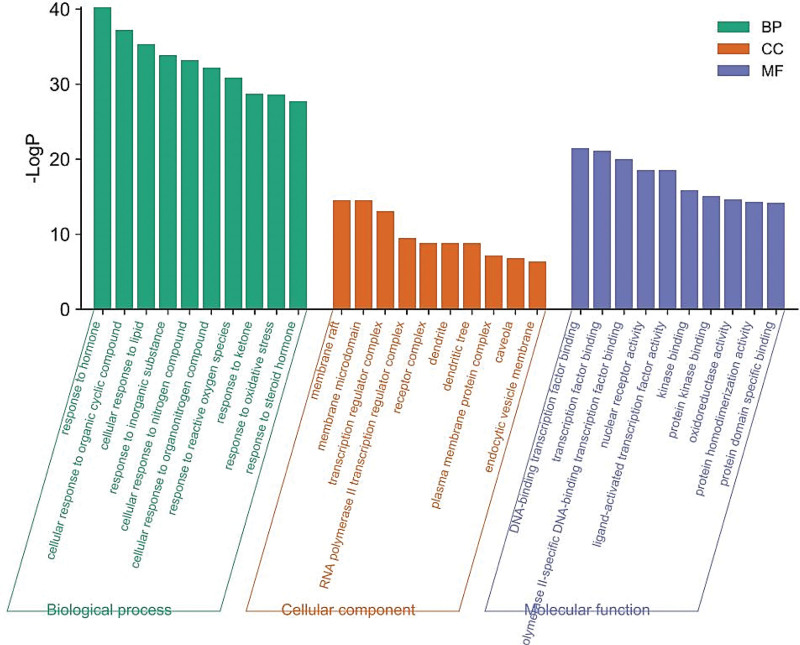
The bar diagram of GO enrichment analysis. GO = gene ontology.

Figures 5 and 6 showed the most significant 10 results of GO functional enrichment analysis in terms of biological process (BP), cellular component (CC), and molecular function (MF), respectively. These 1514 relevant BPs primarily focused on cellular responses to various substances, including hormone, organic cyclic compound, inorganic substance, and lipid, etc. Consistently, the results of GO analysis suggested that transcription regulator complex (CC), membrane microdomain (CC), membrane raft (CC), and transcription factor binding (MF) of DNA − binding and RNA polymerase II − specific DNA − binding were important ways for THD to affect AR.

**Figure 7.**
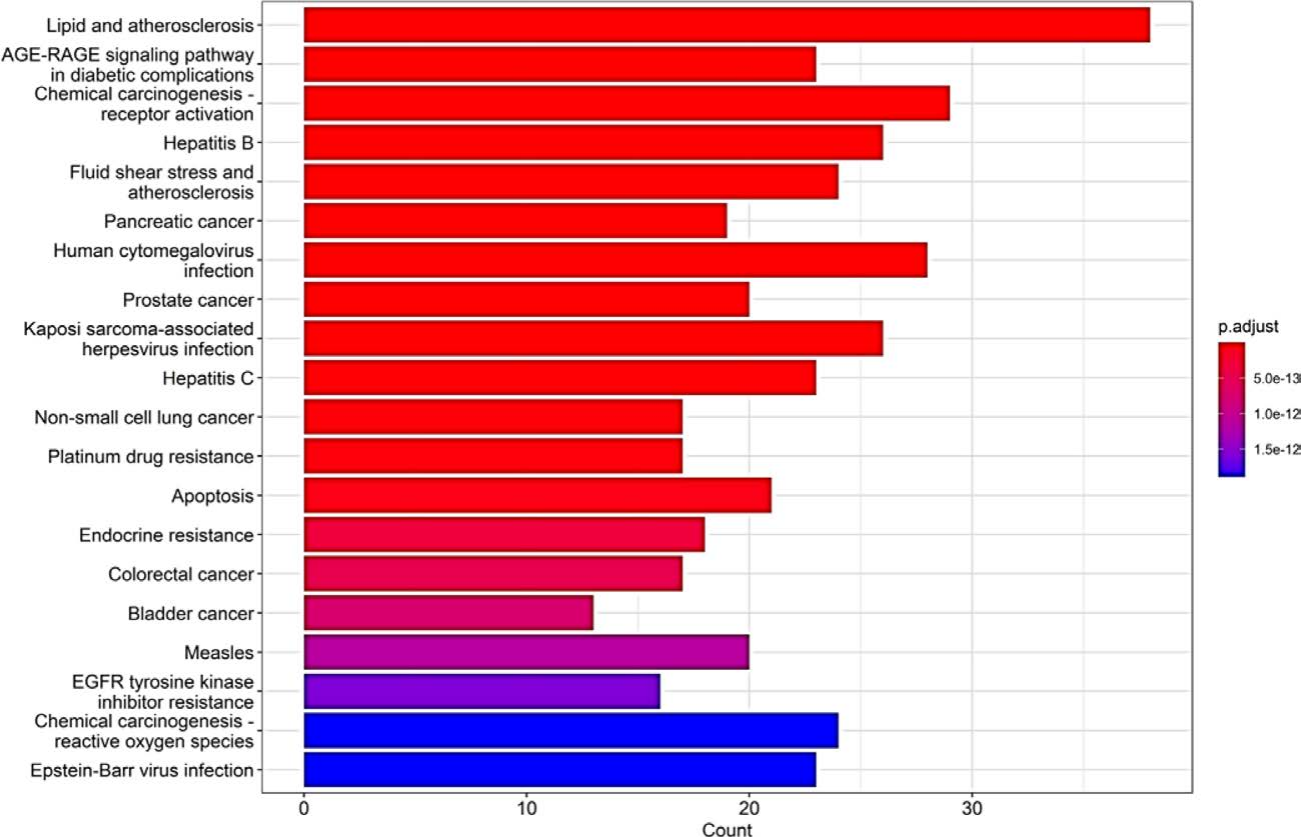
The bar plot of KEGG enrichment analysis. The top 20 items ranked by −log10(*P*) value, gene count, and rich factor. KEGG = kyoto encyclopedia of genes and genomes.

**Figure 8.**
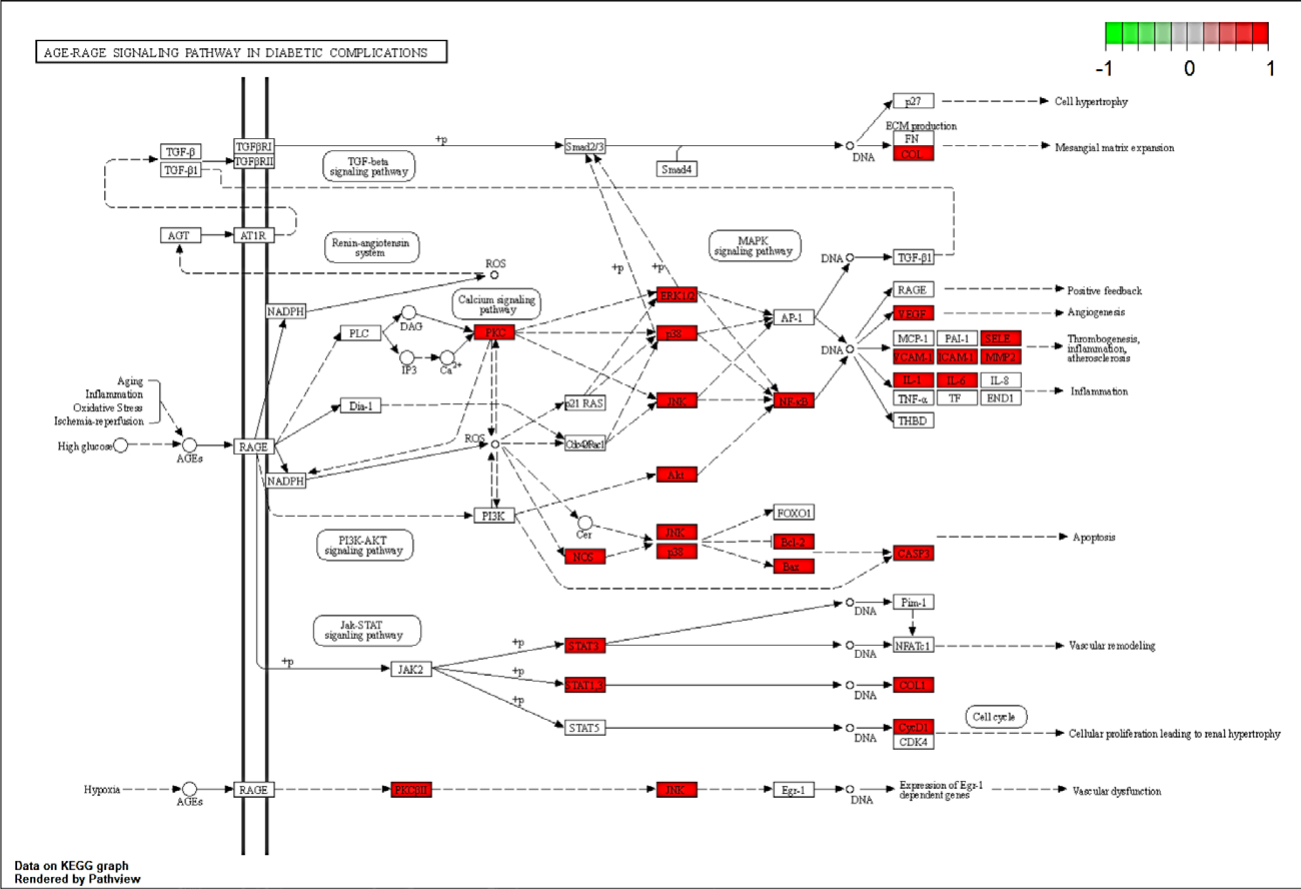
The AGE-RAGE signaling pathway of potential target genes of THD treatment in AR. AR = allergic rhinitis, KEGG = kyoto encyclopedia of genes and genomes, THD = Tongqiao Huoxue decoction.

**Figure 9.**
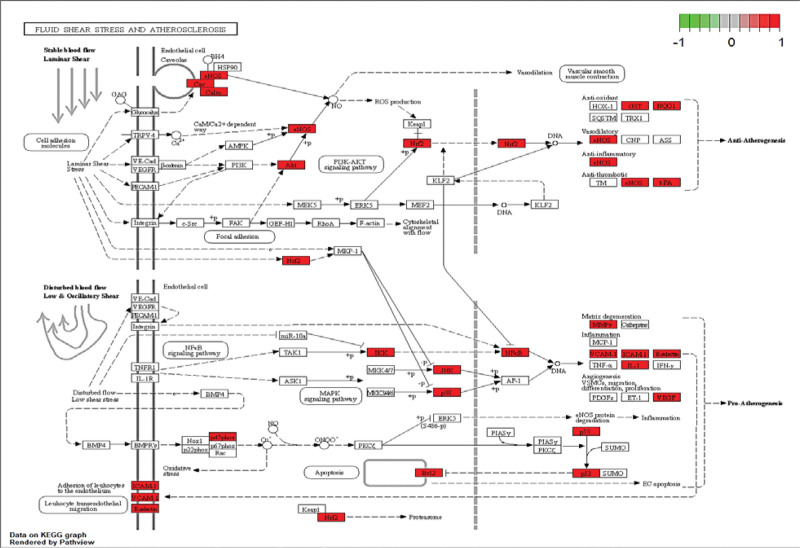
The fluid shear stress and atherosclerosis pathway of potential target genes of THD treatment in AR. AR = allergic rhinitis, KEGG = kyoto encyclopedia of genes and genomes, THD = Tongqiao Huoxue decoction.

The 151 proteins further resulted in 190 KEGG pathways, and the top 20 critical signaling pathways were shown in Figure 7. KEGG pathway enrichment analysis identified these mechanism-related pathways, such as lipid and atherosclerosis, chemical carcinogenesis, AGE-RAGE signaling pathway in diabetic complications, receptor activation, fluid shear stress and atherosclerosis, and human cytomegalovirus infection. Additionally, AGE-RAGE signaling pathway and fluid shear stress and atherosclerosis will be explored further as crucial pathways (Figures 8 and 9).

### 3.6. Validation of molecular docking

**Figure 10.**
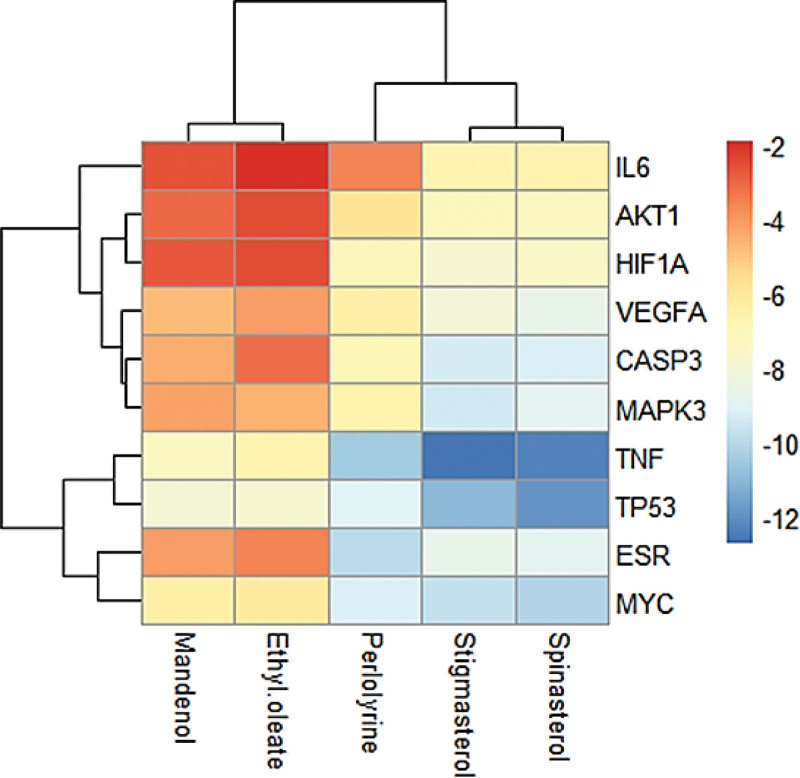
Heat map of binding capacity between key targets and the bioactive compounds.

**Figure 11.**
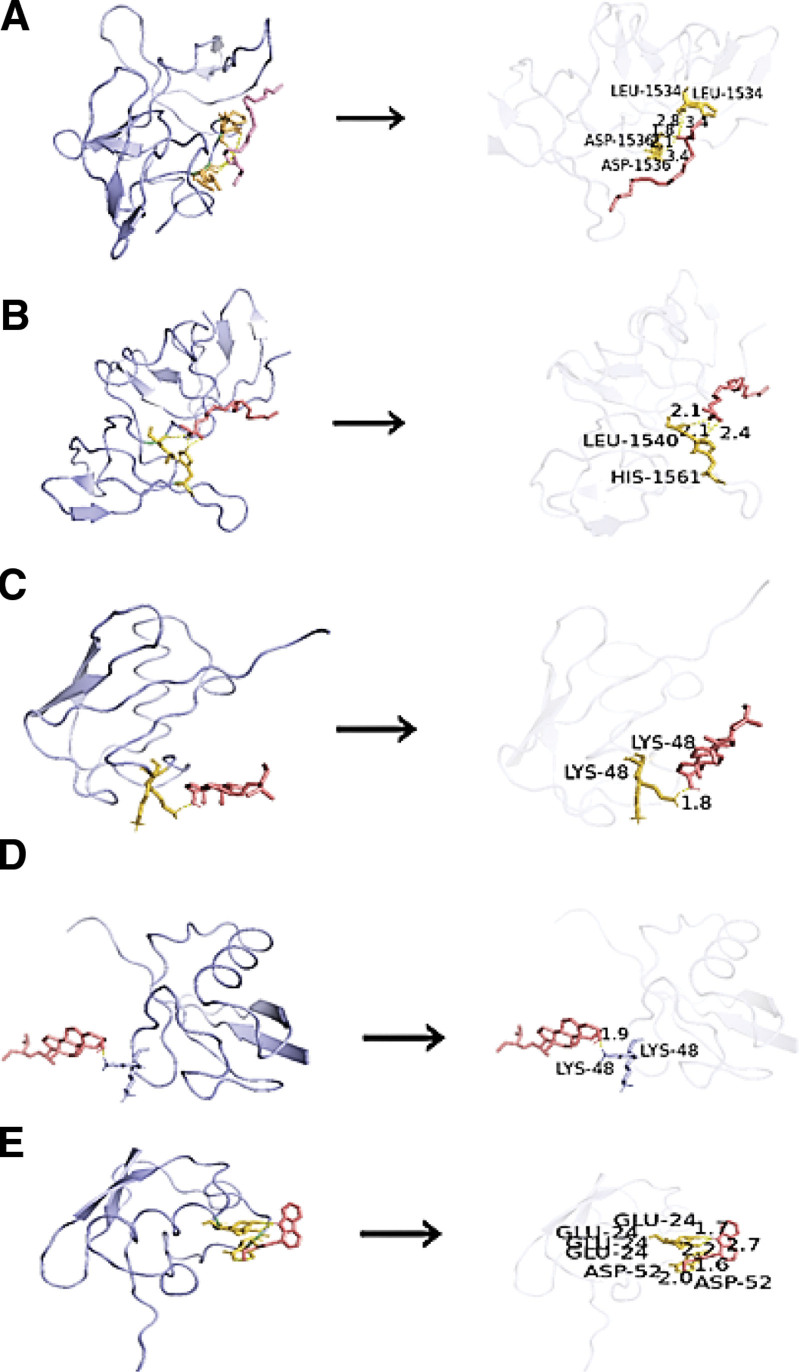
Molecular models of the binding of (A) TP 53 with mandenol, (B) TP53 with ethyl oleate, (C) TNF with spinasterol, (D) TNF with stigmasterol, (E) TNF with perlolyrine. TNF = tumor necrosis factor.

The 10 core target gene-encoded proteins in PPI network, containing AKT1, TP53, IL6, TNF, CASP3, MAPK3, HIF1A, VEGFA, ESR1, and MYC, were docked with five active components respectively by AutoDockTool 1.5.7 software. The binding energy of each component and the protein was obtained, and the heat-map plot was drawn in Figure 10. The binding energies were lower than −5 kcal/mol, indicated solid binding effects. According to the predicted results from molecular docking, spinasterol has the strongest molecular docking ability, and its docking ability with TNF was particularly prominent. The complex produced of each compound with its least energy-efficient protein was visualized using PyMOL software as in Figure 11.

## 4. Discussion

AR is a very common disorder that occurs especially in adolescents. Although AR is not a serious illness, it is clinically relevant because it underlies many complications, is a major risk factor for poor asthma control, and not only is detrimental to health but also has societal costs without effective treatment.^[14]^ When exposed to allergens, IgE acts on mast cells, which in turn release a series of cytokines such as histamine, tumor necrosis factor-α (TNF-α), and leukotriene, resulting in further aggravation of AR.^[15]^ The THD mentioned in this study has been shown to be effective in controlling the symptoms of AR and reducing the number of AR attacks in patients. The theoretical concepts of network pharmacology are coincide with the multi-components and multi-targets of TCM, it can serve as a significant method to analyze drugs, components, and diseases targets.

In this study, a total 232 compounds of THD were found in the TCSMP platform, ethyl oleate (NF), mandenol, perlolyrine, spinasterol, and stigmasterol were identified as core active compounds. Mandenol (ethyl linoleate) by down-regulating inducible nitric oxide syntheses and cyclocxygenase-2 expression and thereby reducing nitric oxide, TNF-α, interleukin-1β, interleukin-6, and prostaglandin E2 production induced by macrophages, inhibited inflammatory activity.^[16]^ In addition to exerting anticoagulant effects by increasing the content of cAMP and cGMP in platelets and relieving platelet aggregation, perlolyrine could also combat the vasoconstriction effect of posterior pituitary hormones.^[17]^ Spinasterol possessed several important pharmacological properties for example anti-inflammation, antiulcer, neuroprotection and anti-pain. And its ant-inflammatory mechanisms included inhibition of cyclooxygenases, antagonism of TRPV1 receptor and attenuation of proinflammatory cytokines and mediators.^[18]^ There is research found that stigmasterol reduced the release of inflammatory factors and the level of oxidative stress induced by interleukin-1β through sterol regulatory element binding transcription factor 2, which indicated the protective effect of stigmasterol on cells.^[19]^ Ethyl oleate could enhance drug OB by both elevating drug solubility and promoting lymphatic transport.^[20]^

Subsequently, 151 intersective target genes of THD and AR were obtained. Base on the PPI network analysis and topology algorithms in Cytoscape software, AKT1, TP53, IL6, TNF, CASP3, MAPK3, HIF1A, VEGFA, ESR1, and MYC were considered the hub target proteins in the treatment of AR. The activation of PI3K/AKT/mTOR pathway promotes abduction autophagy of macrophages.^[21]^ The study of Xiaohan et al^[22]^ confirmed that AKT1 was closely related to cell proliferation and AR could be alleviated by activating AKT1. MYC as a cancer-causing gene expresses three proteins: MYC-1, MYC-2, and MYC-3. The MYC proteins were involved in the regulation of a variety of cellular processes including cell growth, cell cycle, differentiation, apoptosis, angiogenesis, and metabolism et al.^[23]^ In the Bioinformatics analysis of nasal epithelial cell gene expression in seasonal and perennial allergic rhinitis founds that AR patients allergic to house dust mite had significant down-regulation of MYC.^[24]^ The variation of TP53 in amino acid at codon 72 promotes mucous cell hyperplasia by reducing Bcl-2 mRNA half-life and stability.^[25]^ TNF was an important cytokine in the pathology of AR that TNF-α disrupted tight junctions of human airway epithelium and promotes inflammatory cytokines release and TNF-α triggers airway constriction, hyperresponsiveness, and sputum neutrophilia.^[26]^ ESR contains ERα, ERβ, and G protein-coupled estrogen receptor (GPER), of which GPER was significant in immune regulation.^[27]^ It has been demonstrated that GPER-specific receptor agonist G-1 could attenuated the symptoms of AR mice and inhabited the TH2 cell associated inflammatory response by decreasing the level of IL-4, IL-5, and IL-13, and elevating Treg cells proportion and the specific cytokines IL-10 and Foxp3 transcription factor.^[28]^ MAPK3 was one of the MAPK family effectors could regulate the expression of related genes, the transcription, and translation of inflammatory factors.^[29]^

GO annotation and pathway enrichment analyses showed that the major biological processes contained response to hormone, cellular response to organic cyclic compound, cellular response to lipid, membrane raft, membrane microdomain, DNA-binding transcription factor binding, and nuclear receptor activity et al And indicated it may be possible to delay the development of AR by preventing these biological processes form taking place. In the KEGG enrichment analysis of hub genes, the effect of THD against AR may be related to lipid and atherosclerosis, AGE-RAGE signaling pathway in diabetic complications, and fluid shear stress and atherosclerosis. The activation of AGE-RAGE signaling pathway led to increased RAGE expression and oxidative stressors, and blockaded of RAGE signal transduction may be a key measure for the prevention of deleterious consequences of oxidative stress, particularly in chronic disease.^[30]^ After RAGE activation, the elevate of reactive oxygen species (ROS) products and then the oxidative stress response was activated, which means that numerous intracellular structures, such as cellular membranes, proteins, lipids, and DNA were disrupted.^[31]^ Endothelial response to fluid shear stress (FSS) is a critical element of vascular homeostasis.^[32]^ Multi-directional and chaotic FSS caused curved and branched arteries more and this areas with unstable flow were more susceptible to infection and inflammation.^[33]^

The binding mode and binding capacity of active components in THD to potential targets in AR were probed by molecular docking. Our results showed that the active compounds in THD had low binding energy and high binding capacity to TNF, TP53, ESR, and MYC, and the docking scores were mostly < -5kcal/mol which indicated that these protein may be potential binding targets of compounds.

It is necessary to state the present several limitations:Firstly, our conclusion was based on these reviewed and predicted data from online databases, this may have led our results being incomplete without unproven and undocumented compounds or targets. Secondlym absorption pathways, metabolic forms of bioactives in THD should be studied. Lastly, our study could only initially explained the mechanism of THD to treat AR based on the theoretical results of network pharmacology and molecular docking. The experiments in vivo and vitro were needed to further verify the main regulatory targets and pathways of action about THD’s treatment in AR.

## 5. Conclusion

In summary, our analysis identified TNF, TP53, and MYC as central genes associated with THD anti-AR and validated the reliability of predicted central genes using molecular docking techniques. These hub relevant targets may through its function of regulating cell growth, cycle, apoptosis, and angiogenesis, promoting mucous cell hyperplasia, and decreasing inflammatory cytokines release and through AGE-RAGE signaling pathway in diabetic complications, and fluid shear stress and atherosclerosis pathway to played its role in relieving inflammatory, anti-oxidative stress response, and promoting cell proliferation to improve AR. This conclusion proved that THD had the characteristics of combined action of multi-targets and multi-pathways, and provided new directions for exploring the potential mechanism of THD’s treatment in AR. However, our study had some certain deficiencies and the hub genes and its pharmacological mechanism of THD in the treatment of AR still need to be validated by in vitro and in vivo experiments.

## Author contributions

**Conceptualization:** Jiani Wu.

Funding acquisition: Zukang Qiao.

Investigation: Zukang Qiao.

Methodology: Fang Zhang.

Resources: Fang Zhang.

Software: Jiani Wu.

Supervision: Zukang Qiao.

Visualization: Fang Zhang.

Writing – original draft: Qu Shen.

Writing – review & editing: Zhiling Che.

## References

[1] Zhengqing L Jiajia R Jirong Z. Association between IL1RL1 gene polymorphisms and allergic rhinitis risk in the Chinese Han population. J Clin Lab Anal. 2022;2022:e24747. 10.1002/jcla.24747 PMC9701900 36310516

[2] Kaiyun P Guodong L Mouhan L. Prevalence and risk factors for allergic Rhinitis in China: a systematic review and meta-analysis. Evid Based Complement Alternat Med. 2022;2022:7165627. 36193147 10.1155/2022/7165627 PMC9525776

[3] Holger H. TLRs, NLRs and RLRs: innate sensors and their impact on allergic diseases - a current view. Immunol Lett. 2011;139:14–24. 21554901 10.1016/j.imlet.2011.04.010

[4] Bousquet J Anto J Bachert C. Allergic rhinitis. Nat Rev Dis Primers. 2020;3:95. 10.1038/s41572-020-00227-0 33273461

[5] Wu AC Dahlin A Wang A. The role of environmental risk factors on the development of childhood allergic rhinitis. Children (Basel). 2021;8:708. 34438599 10.3390/children8080708 PMC8391414

[6] Diana Q Abraham A Yaicith A. Validation of the Spanish language version of the control of allergic rhinitis and asthma test. NPJ Prim Care Respir Med. 2022;32:47. 36309520 10.1038/s41533-022-00313-8 PMC9617860

[7] Yifan M Chengshuo W Luo Z. Recent developments and highlights in allergic rhinitis. Allergy. 2019;74:2320–8. 31571226 10.1111/all.14067

[8] Naclerio R Ansotegui I Bousquet J. International expert consensus on the management of allergic rhinitis (AR) aggravated by air pollutants: Impact of air pollution on patients with AR: current knowledge and future strategies. World Allergy Organ J. 2020;13:100106. 32256939 10.1016/j.waojou.2020.100106 PMC7132263

[9] Reitsma S Subramaniam S Fokkens W. Recent developments and highlights in rhinitis and allergen immunotherapy. Allergy. 2018,73:2306–2313. 30260494 10.1111/all.13617

[10] ShengKang H YuLing H YuanShiun C. Prescriptions of traditional Chinese medicine, western medicine, and integrated Chinese-Western medicine for allergic rhinitis under the National Health Insurance in Taiwan. J Ethnopharmacol. 2015,173:212–6. 26172981 10.1016/j.jep.2015.06.051

[11] Rose-John S. Interleukin-6 family cytokines. Cold Spring Harb Perspect Biol. 2018;10:a028415. 28620096 10.1101/cshperspect.a028415 PMC5793756

[12] Hongen C Na Z Hongwei A. Effect of Tongqiao Huoxue decoction on serum TNF-α and IL-6 levels after cerebral ischemia-reperfusion injury in rats. J Cengdu Univ Tradit Chin Med. 2018;3:18–20.

[13] Zhou Z Chen B Chen S. Applications of network pharmacology in traditional Chinese medicine research. Evid Based Complement Alternate Med. 2020;14:1646905. 10.1155/2020/1646905 PMC7042531 32148533

[14] Greiner AN Helling PW Rotiroti G. Allergic rhintis. Lancet. 2012;378:2112–22. 10.1016/S0140-6736(11)60130-X 21783242

[15] Lotfi R Davoodi A Mortazavi SH. Imbalanced serum levels of resolvin E1 (RvE1) and leukotriene B4 (LTB4) in patients with allergic rhinitis. Mol Biol Rep. 2020;10:7745–54. 10.1007/s11033-020-05849-x 32960415

[16] Park SY Seetharaman R Ko MJ. Ethyl linoleate from garlic attenuates lipopolysaccharide-induced pro-inflammatory cytokines production by inducing heme oxygenase-1 in RAW264.7 cells. Int Immunopharmacol. 2014;19:253–61. 24508058 10.1016/j.intimp.2014.01.017

[17] Chunlong R Xinjie Z Wenhui L. Exploration of potential molecular mechanism of Chuanxiong in treatment of tension-type headache based on network pharmacology and molecular docking. Chinese Pharmacol Bull. 2022;38:140–7.

[18] Majeed M Ahmad F Mundkur L. Pharmacology of α-spinasterol, a phytosterol with nutraceutical values: a review. Phytother Res. 2022;36:3681–90. 35802356 10.1002/ptr.7560

[19] Mo Z Xu P Li H. Stigmasterol alleviates interleukin-1β-induced chondrocyte injury by down-regulatingsterol regulatory element binding transcription factor 2 to regulateferroptosis. Bioengineered. 2021;12:9332–40. 34806937 10.1080/21655979.2021.2000742 PMC8810005

[20] Qiao X Jia S Xiuhua Y. Microemulsions containing long-chain oil ethyl oleate improve the oral bioavailability of piroxicam by increasing drug solubility and lymphatic transportation simultaneously. Int J Pharm. 2016;511:709–18. 27473280 10.1016/j.ijpharm.2016.07.061

[21] Ruijun S Xiaoting J Weifang Z. Particulate matter exposure induces the autophagy of macrophages via oxidative stress-mediated PI3K/AKT/mTOR pathway. Chemosphere. 2017;167:444–53. 27750168 10.1016/j.chemosphere.2016.10.024

[22] Xiaohan W Baoping Z Xiao L. Higenamine alleviates allergic rhinitis by activating AKT1 and suppressing the EGFR/JAK2/c-JUN signaling. Phytomedicine. 2021;86:153565. 33945919 10.1016/j.phymed.2021.153565

[23] Beaulieu ME Castillo F Soucek L. Structural and biophysical insights into the function of the intrinsically disordered Myc oncoprotein. Cells. 2020;9:1038. 32331235 10.3390/cells9041038 PMC7226237

[24] Sun LW Liu ZY Sha JC. Bioinformatics analysis of nasal epithelial cell gene expression in seasonal and perennial allergic rhinitis. Zhonghua Er Bi Yan Hou Tou Jing Wai Ke Za Zhi. 2022;57:425–32. 35527433 10.3760/cma.j.cn115330-20210630-00397

[25] Taeeew D Fort S Mebratu Y. Effects of wood smook constituents on mucin gene expression in mice and human airway epithelial cells and on nasal epithelia of subjects with a susceptibility gene variant inTp53. Environ Health Perspect. 2022;103:17010. 10.1289/EHP9446 PMC8785869 35072516

[26] Steelant B Seys SF Van G. Histamine and T helper cytokine-driven epithelial barrier dysfunction in allergic rhinitis. J Allergy Clin Immunol. 2018;141:951–63. 29074456 10.1016/j.jaci.2017.08.039

[27] Pelekanou V Kampa M Kiagiadaki F. Estrogen anti-inflammatory activity on human monocytes is mediated through cross- talk between estrogen receptor ERα36 and GPR30/GPER1. J Leukoc Biol. 2016;99:333–47. 26394816 10.1189/jlb.3A0914-430RR

[28] Yunxiu W Zhaowei G Liying H. The environmental hormone nonylphenol interferes with the therapeutic effects of G protein-coupled estrogen receptor specific agonist G-1 on murine allergic rhinitis. Int Immunopharmacol. 2020;78:106058. 31835084 10.1016/j.intimp.2019.106058

[29] Jia Y Zou Wang Y. Mechanism of allergic rhinitis treated by Centipeda minima from different geographic areas. Pharm Biol. 2021;59:606–18. 34010591 10.1080/13880209.2021.1923757 PMC8143626

[30] Daffu G del Pozo CH O’Shea KM. Radical roles for RAGE in the pathogenesis of oxidative stress in cardiovascular diseases and beyond. Int J Mol Sci. 2013;14:19891–910. 24084731 10.3390/ijms141019891 PMC3821592

[31] Kay AM Simpson CL Stewart JA. The role of AGE/RAGE signaling in diabetes-mediated vascular calcification. J Diabetes Res. 2016;6809703. 27547766 10.1155/2016/6809703 PMC4980539

[32] Kant S Tran KV Kvandova M. PGC1αRegulates the endothelial response to fluid shear stress via telomerase reverse transcriptase control of heme oxygenase-1. Arterioscler Thromb Vasc Biol. 2022;42:19–34. 34789002 10.1161/ATVBAHA.121.317066 PMC8702461

[33] Baeyens N. Fluid shear stress sensing in vascular homeostasis and remodeling: towards the development of innovative pharmacological approaches to treat vascular dysfunction. Biochem Pharmacol. 2018;158:185–91. 30365948 10.1016/j.bcp.2018.10.023

